# Fungal Keratitis, Epidemiology and Outcomes in a Tropical Australian Setting

**DOI:** 10.3390/tropicalmed9060127

**Published:** 2024-06-03

**Authors:** Leah N. Kim, Hema Karthik, Kate Elizabeth Proudmore, Sarah Elizabeth Kidd, Robert William Baird

**Affiliations:** 1 Ophthalmology Department, Royal Darwin Hospital, 105 Rocklands Drive, Tiwi, NT 0811, Australia; 2 Territory Pathology, Royal Darwin Hospital, 105 Rocklands Drive, Tiwi, NT 0811, Australia; 3 National Mycology Reference Centre, Microbiology & Infectious Diseases, SA Pathology, Frome Road, Adelaide, SA 5000, Australia

**Keywords:** tropical, fungal, keratitis

## Abstract

Background: Fungal keratitis is an ophthalmic emergency that can cause visual impairment and blindness. We reviewed the epidemiology and clinical features of fungal keratitis in a tropical Australian setting. Objectives: To document the clinical and microbiological characteristics of fungal keratitis in an Australian tropical setting. Methods: A retrospective cohort study of patients with fungal keratitis from October 2014 to December 2022 was conducted at Royal Darwin Hospital, Northern Territory, Australia. We reviewed all patients with culture-proven fungal keratitis and their outcomes. Results: There were 31 patients identified. Aboriginal and Torres Strait Islander (ATSI) patients were of a significantly younger median age (28 years) compared to non-ATSI patients (42 years), and they also presented later to health care. Contact lens use and ocular trauma were the most common predisposing factors. Most patients presented with a corneal infiltrate and corneal epithelial defect, and the central visual axis was affected in 54% of patients. *Curvularia* spp. and *Fusarium* spp. were the commonest causative fungi (39% and 30% respectively). Conclusions: Our series is different and reveals a wider range of fungal species identified over the 7 years of the study, in particular, a range of *Curvularia* spp. were detected. Access to eye health services in rural and remote settings is important, particularly for ATSI patients, as morbidity remains high.

## 1. Introduction

Fungal keratitis is an ophthalmic emergency that can cause significant visual impairment and blindness. It is diagnosed based on slit lamp microscopy findings and positive fungal culture from corneal scrapings. Clinically, fungal keratitis may appear as corneal ulcers with white-gray infiltrates and feathery margins, and there may be satellite lesions. Risk factors for this condition include prolonged contact lens use, topical steroid eye drop use, ocular trauma with vegetative matter, and ocular surface disease. Management of fungal keratitis can often be challenging due to delays in diagnosis and variable response to treatment. This is especially the case where eye health services are limited in rural and remote settings, leading to delayed presentations, more extensive corneal disease at initial presentation, and poorer outcomes [1].

There are geographic variations in the prevalence and characteristics of fungal keratitis. One review estimated that between 1 and 1.4 million new cases of fungal keratitis occur each year [1] worldwide. The estimated annual incidence ranged from 73 per 100,000 in South Asia to just 0.02 per 100,000 in Europe [2]. Recent Australian data [3] reviewed the seasonal and geographic variations in the incidence of fungal keratitis in tropical Queensland. Overall, the cumulative incidence per 100,000 population was 4.7, but it was significantly higher in tropical latitudes, at 7.9 per 100,000 population. The most commonly identified causative fungi were *Fusarium* spp. (33%) and *Aspergillus* spp. (13%); however, worldwide, over 100 different fungal species have been associated with the condition [1]. Fungal taxonomy and nomenclature have undergone significant changes over the past 10–15 years as genomic phylogenetic analyses augment traditional phenotypic characterisation, leading to changes in the nomenclature and species of organisms associated with different infection types [4].

In clinical practice, a combination of antifungals is often used to treat fungal keratitis due to its complexity and its potential to have visually devastating outcomes. Natamycin 5% is the only FDA-approved topical antifungal medication, but it is not always available outside of tertiary centres. The Mycotic Ulcer Treatment Trial 1 [5] demonstrated that topical natamycin 5% had better outcomes than topical voriconazole 1% in filamentous fungal keratitis. Additionally, the Mycotic Ulcer Treatment Trial 2 [6] showed that adding oral voriconazole to a regimen of both topical natamycin 5% and voriconazole 1% did not provide any therapeutic benefit in cases of severe filamentous fungal ulcers.

The Top End of the Northern Territory (TENT) encompasses the northernmost section of Australia, covering an area in excess of 500,000 km^2^ with a sparse population of 180,000 and a distinct tropical monsoonal season [7]. Aboriginal and Torres Strait Islanders (ATSI) account for 31% of the NT population [8]. In tropical Australia, Richards et al. [9] completed the only study to date that has characterised both bacterial and fungal keratitis in the Northern Territory. In this study, there were 13 cases of fungal keratitis between 2007–2014 presenting to Royal Darwin Hospital. The most common mould isolated was *Curvularia* spp. Fungal keratitis was also not significantly more common in Indigenous patients or in patients with reported trauma. Our study further examines cases of culture-proven fungal keratitis in TENT in more recent years. Understanding the profile of fungal keratitis presentations will assist in its future management.

## 2. Materials and Methods

This was a retrospective cohort study of all patients diagnosed with fungal keratitis from October 2014 to December 2022 at Royal Darwin Hospital, Northern Territory, Australia. Fungal isolates from corneal specimens taken in this period were identified by reviewing the clinical microbiology records of Royal Darwin Hospital laboratory information system: Labtrak (InterSystems, Cambridge, MA, USA). Demographic and clinical data were then retrieved from the clinical records from the Department of Medical Records, Royal Darwin Hospital. Demographic data included patient age, sex, and residential area. Residential areas were classified according to the Australian Statistical Geography Standard Remoteness Areas Scale from the Australian Bureau of Statistics: Zone 1, major cities of Australia; Zone 2, inner regional Australia; Zone 3, outer regional Australia; Zone 4, remote Australia; and Zone 5, very remote Australia. Clinical data included the patient’s visual acuity as measured on the Snellen chart, risk factors for fungal keratitis, findings of their corneal exam, and treatment details.

The cornea is non-sterile and not at physiological temperature. Fungal detection was by microscopy and culture. Fungal cultures were specifically performed using Sabouraud’s dextrose agar (Thermo Fisher Scientific, Waltham, MA, USA) with up to 6 weeks of incubation at 25 °C. Fungi typically grow optimally on Sabouraud’s dextrose agar at this lower incubation temperature compared to the temperature used to culture bacterial pathogens. Gram stains typically detect yeasts, but the hyphae of moulds may not stain well or may be missed at 1000× magnification. Speciation and susceptibility testing was performed at the National Mycology Reference Centre, SA Pathology (Adelaide, Australia) using the Sensititre YeastOne panel (Thermo Fisher Scientific, Waltham, MA, USA), either YO10 or AUSNMRC1 with isavuconazole, which has demonstrated utility for a range of moulds. Results were reported as a minimum inhibitory concentration (MIC) or, for anidulafungin, minimum effective concentration (MEC). At the time of writing, the polyene agent natamycin is not commercially available in Australia for in vitro susceptibility testing purposes. Many fungal species, including those in this study, do not have clinical breakpoints for the available antifungal drugs. Patient details, including epidemiology and clinical data, were retrieved from the clinical records from the Department of Medical Records, Royal Darwin Hospital, Rocklands Drive Tiwi, Northern Territory.

### Outcomes

Patient outcomes were grouped as follows: good outcome—final visual acuity (VA) of 6/12 or better, no complications, no surgical intervention, no decrease in VA during treatment; moderate outcome—final VA 6/15–6/60, no complications, no surgical intervention, no decrease in VA during treatment; poor outcome—final VA < 6/60 or decrease in VA during treatment, developed complications of infection, required surgical intervention.

Descriptive statistics summarised the socio-demographic, clinical characteristics, pathology results, antimicrobial therapy, and outcomes. Descriptive analyses were performed using Microsoft Excel Version 2012. Statistical analysis was by Fisher’s exact test for categorical variables. The authors confirm that the ethical policies of the journal have been adhered to and the appropriate ethical review committee approval was received: ethics approval HREC 2020–3854 was obtained from the NT Human Research Ethics committee.

## 3. Results

Thirty-one patients with fungal keratitis between October 2014 to December 2022 were identified, an annual incidence of 2.2 cases/100,000 in the Top End population. The median age was 34 years (IQR 26–48 years), 58% were male, and 26% were ATSI patients. The demographic details of the patients categorised by ATSI status are presented in Table 1. ATSI patients were more likely to live in remote and very remote Australian locations of TENT (*p* < 0.003) and were of a younger median age compared to non-ATSI patients (28 years vs. 42 years, respectively). Contact lens use was the most common predisposing risk factor overall (45%), but keratitis associated with use of contact lenses was confined to non-ATSI patients. This was followed by ocular trauma (32%). Other relevant risks factors included a history of cleaning or playing in dirt, and a patient with keratoconus in acute psychiatric crisis. There were no recorded predisposing risk factors for 16% of patients.

Patients were referred from general practice (number (n) = 10, 30%), the emergency department (n = 16, 52%), an optometrist (n = 1, 3%), or not recorded (n = 2, 12%). They were all seen on the same day as the referral. The median time to presentation for ophthalmic care from the date of onset of symptoms was 4 days (IQR 1–6). The median time to presentation was 6 days (IQR 4.5–7.5) for ATSI patients and 2 days (IQR 1–5.5) for non-ATSI patients. Fourteen patients were admitted with a median hospital admission duration of 9 days (IQR 5–16), of which 5 patients were of ATSI background.

The left eye was more commonly involved (62% of patients) than the right, and one patient had bilateral keratitis. Visual acuity at presentation was 6/12 or better in 65% of patients. ATSI patients presented with VA 6/12 or better in 57% (n = 4) and VA < 6/60 in 43% (n = 3). Non-ATSI patients presented with VA 6/12 or better in 68% (n = 13), VA 6/15–6/60 in 16% (n = 3) and VA < 6/60 in 16% (n = 3). A corneal epithelial defect was present in 20 out of 24 cases (83%), with a median area of 5.4 mm^2^ (IQR 2.0–15.8). An infiltrate was present in 21 out of 24 patients (87.5%), with a median area of 3.4 mm^2^ (IQR 1.4–12.5). The central visual axis was involved in 13 out of 24 patients (54%). Hypopyon was present in 4 out of 26 cases (15%), corneal thinning in 7 out of 21 cases (33%), satellite lesions in 4 out of 21 cases (19%), and ring infiltrates in 1 out of 20 cases (5%).

The most common agents used for therapy were topical natamycin and topical or oral voriconazole. Topical natamycin was used as a single antifungal therapy in 8 cases (44%); combined with oral voriconazole in 3 cases (17%); combined with topical voriconazole in 1 case (6%); or combined with both oral and topical voriconazole in 2 cases (12%). Topical voriconazole was used as a single antifungal therapy in 1 case (6%) or combined with oral voriconazole in 3 cases (17%). There was also a case that was treated with topical natamycin, propamidine isethionate (Brolene), and polyhexamethylene biguanide. Table 2 describes the treatment given, categorised by fungal species. The median treatment duration was 3 months (IQR 1–3).

The cultured fungi yielded a wide diversity in genera and species, with high rates of *Fusarium* (9 isolates) and *Curvularia* (12 isolates) species. Other fungi included *Massarina* spp., *Penicillium chrysogenum*, *Phoma* spp., *Purpureocillium lilacinum*, *Pyrenochaeta* spp., *Quambalaria cyanescens*, *Rhodotorula mucilaginosa*, *Aureobasidium pullulans*, and *Colletotrichum queenslandicum*. Table 3 presents the details of the fungal isolates from keratitis patients and MIC data.

The 12 *Curvularia* species isolated included *Curvularia lunata* (5 isolates), *C. brachyspora* (1), *C. geniculata* (1), and *C. pallescens* (3), and two non-speciated *Curvularia* isolates, reflecting the increasing taxonomic changes in the genera. The wide range of other moulds isolated reflects the expanding mold taxonomy. *Purpureocillium lilacinum* was previously classified within the genus Paecilomyces and is commonly isolated from soil, decaying vegetation, insects, and nematodes. It is also a causative agent of infection in humans and other vertebrates. One patient had both *Penicillium chrysogenum* and an *Aspergillus* spp. detected, but the *Aspergillus* spp. was not considered to be the pathogen in this case and more likely represented a laboratory contaminant. *Aspergillus* spp. were not otherwise detected in this study.

Table 3 details the fungal species and available MIC data for a range of antifungal agents. Very few antifungal agents have interpretative criteria established for differing fungal species. Fungal isolates with low MIC levels that are below measurable blood and body fluid antifungal concentrations may be used in discussion with pharmacists, microbiologists, and infectious disease physicians.

Outcome data are presented in Table 4. Most patients (54%) had a good outcome. The number of days taken for an associated epithelial defect to healing ranged from 1 to 80 days, with a median time of 7 days (IQR 4–12.5). Nine patients were lost to follow-up in our study. Five patients did not attend a follow-up clinic or were transferred to another facility. There were no cases of endophthalmitis recorded.

## 4. Discussion

Our study highlights some unique demographic and microbiological features of fungal keratitis in the Northern Territory. Firstly, ATSI patients presented at a significantly earlier median age of 28 years and had a delayed presentation of 6 days compared to non-ATSI patients (median age 42 years, presentation at 2 days). ATSI patients also had trauma as a common predisposing factor (38%), whereas non-ATSI patients and contact lens use were strongly associated (61%). Most of our ATSI patients lived in remote and very remote areas, which was a contributing factor to their delayed presentation. While refractive errors are common in ATSI patients, eye health services are less common in remote and very remote areas, making contact lens use less common in this population. Overall however, the median age of all patients (34 years) with a slight male predominance (56%) was similar to a previous study from tropical North Queensland [3]. One of the other notable observations in our study was the wide variety and differences in fungal pathogens when compared to other Australian single-location series [10]. Fungal keratitis is more common in tropical regions, and most cases have been attributed to *Fusarium* spp. and *Aspergillus* spp. in studies internationally [1] and from Far North Queensland [3], while *Candida* spp. were predominant in Victoria [11]. Our series was different and revealed a wide diversity of fungal species identified over the 8 years of the study, in particular, a wide number and range of *Curvularia* spp. were detected. *Curvularia* spp. were also common in Richards’ previous NT study [9], suggesting it is true reflection of Top End fungal keratitis pathogens.

*Curvularia* is a genus of melanised filamentous fungi commonly found in soil, plants, and decaying organic matter, and most species may be found in tropical regions [12]. While most species are saprophytic (meaning they feed on dead or decaying organic matter), some can also cause opportunistic infections in humans and animals. *Curvularia* spp. have been noted previously as a cause of fungal keratitis, and a series of 43 patients over 30 years was published in 2000 [12]. Interestingly, trauma, usually with plants or dirt, was a risk factor in one half of cases, and 69% occurred during the hot, humid summer months along the US Gulf Coast, mirroring the local tropical Top End climate. A more recent series from India [13] reviewed fungal dematiaceous keratitis between 2017 and 2019 and noted there were 56 (70.8%), 15 (18.9%), and 8 (10.1%) patients with *Curvularia*, *Scedosporium*, and *Alternaria* keratitis, respectively. The conclusions of this study were that larger size of the infiltrate, posterior stromal involvement, and presence of a hypopyon were poor prognostic indicators among all three genera. However, fungal genus was not a predictor of clinical resolution.

Not all tropical microbiology laboratories have extensive mycology facilities, so this difference in fungal species prevalence may reflect local diagnostic capacity; however, *Curvularia* spp., *Aspergillus* spp., and *Fusarium* spp. are morphologically and microscopically distinct entities and have a number of distinguishing microscopic and cultural features. Diagnosis traditionally relies on specialised culture and media, although the development of nucleic acid-based diagnostics may provide additional capacity in areas where traditional diagnostics are not available [1].

Natamycin is the antifungal medication most commonly used in the treatment of fungal keratitis, particularly in cases caused by filamentous fungi. It belongs to the polyene class and works by binding to ergosterol in the fungal cell membrane, leading to disruption of membrane integrity. Natamycin exhibits broad-spectrum activity and is particularly effective against *Fusarium* spp., a common cause of fungal keratitis, as well as *Aspergillus* spp. [14]. Natamycin is typically administered topically as eye drops or ointment and is generally well-tolerated with minimal systemic absorption. Common adverse effects include transient ocular irritation, burning, and a stinging sensation, which typically resolve with continued use. Systemic side effects are rare due to limited absorption. Natamycin has limited corneal penetration, which means that it will primarily act upon fungal organisms residing on the corneal surface and within superficial layers. Therefore, it is most effective for treating superficial fungal keratitis or where there is involvement of the anterior corneal stroma.

Antifungal susceptibility testing could provide valuable information on the susceptibility of fungal isolates to natamycin and help to guide treatment decisions, particularly in cases of refractory or recurrent fungal keratitis. However, MIC results should be interpreted in conjunction with clinical data and other factors, such as corneal penetration of the antifungal agent and patient response to therapy, to optimise treatment outcomes [14]. Additionally, natamycin MIC testing was not available over the course of this study. The wide variety of combinations of topical and oral antifungals received by our patients was similar to that in other ophthalmology centres [15]. Topical natamycin was the most common antifungal used. However the combination of antifungals used varied between patients. Management of fungal keratitis is difficult due to delays in presentation, evolving clinical signs, the time taken for fungal cultures to be processed, and also treatment not being standardised. For instance, fungal keratitis, being relatively rare, does not feature in the Australian Therapeutic Guidelines—Antibiotics [16], a widely used therapy guide for Australian doctors, due to the need for specialist diagnosis and therapy, as compared to bacterial keratitis, which is discussed. Future improved access to telehealth may assist rural and remote management and allow early triage to a specialist centre. Early in vivo confocal microscopy could potentially facilitate an earlier diagnosis. Vaddavalli et al. [17] found confocal microscopy had a sensitivity of 88.3% and specificity of 91.1% in identifying fungal filaments or *Acanthamoeba* spp. cysts as a diagnostic modality. However, this is equipment is not available in our institution and is often unavailable outside tertiary centres.

A limitation of our study is that it is a retrospective chart analysis and therefore only available recorded data could be collected. Not all patients were available for follow-up to review long-term outcomes and make conclusions. Standardised forms could be used in clinical practice to record relevant data on patients with fungal keratitis, which would help future research.

Fungal keratitis is an under-recognised disease with public health consequences. Raising awareness, rural practitioner education, improving access to natamycin, developing a laboratory able to perform natamycin susceptibility testing, and incorporation of fungal keratitis as an entity to be discussed in *Therapeutic Guidelines—Antibiotics* [16] are all steps that could contribute to better recognising and managing this condition.

## Figures and Tables

**Table 1:** Demographic characteristics of patients with fungal keratitis categorised by ATSI status.

Parameter	ATSI	Non-ATSI	Total	*p*-Value
Number of patients (%)	8 (26)	23 (74)	31 (100)	
Median age (years)	28 (IQR 8–32)	42 (IQR 29–48)	34 (28–48)	0.047
Sex–male (%)	4 (50)	14 (61)	18 (58)	
**Residence**				
Zone 1, 2, 3 (%)	0 (0)	21 (91)	21 (68)	0.003
Zone 4 (%)	1 (12)	1 (4)	2 (6)	
Zone 5 (%)	7 (88)	1 (4)	8 (26)	
**Predisposing risk factors**				
Contact lens use (%)	0 (0)	14 (58)	14 (45)	0.04
Ocular trauma (%)	3 (38)	7 (29)	10 (32)	
Other (%)	2 (25)	1 (4)	3 (10)	
None (%)	3 (28)	2 (8)	5 (16)	

Note: Abbreviations: ATSI, Aboriginal and Torres Strait Islander; IQR, interquartile range.

**Table 2:** The clinical features and therapy of the keratitis patients categorised by the causative fungal group.

Clinical Finding	*Fusarium* spp.	*Curvularia* spp.	Other	Total
Number of patients	9 (29)	12 (39)	10 (32)	31 (100)
**Visual acuity at presentation**				
6/12 or better (%)	5 (56)	7 (70)	5 (63)	17 (63)
6/15–6/60 (%)	1 (11)	2 (20)	0 (0)	3 (11)
>6/60 (%)	3 (33)	1 (10)	3 (37)	7 (26)
Median epithelial defect area	5.7 (IQR 2.6–15.3)	3.0 (IQR 1.4–15.4)		5.4 (IQR 2.0–15.8)
Median infiltrate area	4.8 (IQR 2.4–9.4)	2.7 (IQR 1.4–13.4)		3.4 (IQR 1.4–12.5)
**Treatment**				
Topical natamycin (%)	4 (44)	2 (17)	2 (20)	8 (26)
Topical natamycin and oral voriconazole (%)	1 (11)	1 (8)	1 (10)	3 (10)
Topical natamycin and topical voriconazole (%)	1 (11)	0	0	1 (3)
Topical natamycin, topical voriconazole, and				
oral voriconazole (%)	2 (22)	0	0	2 (6)
Topical natamycin, propamidine isethionate,	0	0	1 (10)	1 (3)
polyhexamethylene biguanide (%)				
Topical voriconazole (%)	1 (11)	0	0	1 (3)
Topical voriconazole and oral voriconazole (%)	0	1 (8)	2 (20)	3 (10)
Treatment data unavailable (%)	0	8 (67)	4 (40)	12 (39)

**Table 3:** Fungal isolates identified from the keratitis patients, with antifungal MICs (µg/mL).

Fungal Species Isolated	Number (%)	Anidulafungin	5 Flucytosine	Posaconazole	Voriconazole	Itraconazole	Amphotericin B
*Aureobasidium pullulans*	1 (3)	0.25	4	0.25	0.25	0.25	2
*Colletotrichum queenslandicum*	1 (3)	>8	>64	0.5	0.5	1	0.25
*Curvularia*							
*Curvularia lunata*	5 (16)	>8	>64	0.25	0.25–0.5	0.5	0.5
*Curvularia* sp. ^†^	7 (23)	>8	>64	0.03–0.12	0.25–1	0.12–0.25	0.25–1
*Fusarium*							
*Fusarium* sp.	2 (7)	>8	>64	1–2	0.5–2	>16	2
*Fusarium solani complex*	7 (23)	>8	>64	>8	4–8	>16	1–4
*Massarina* sp.	1 (3)	>8	16	>8	2	>16	>8
*Penicillium chrysogenum*	2 (7)	0.25	>64	0.5	0.5	0.5	4–8
*Purpureocillium lilacinum*	1 (3)	0.25	>64	0.5	0.12	0.5	>8
*Pyrenochaeta* species sp.	1 (3)	>8	>64	0.25	0.5	0.25	0.25
*Quambalaria cyanescens*	1 (3)	>8	>64	0.008	0.008	0.015	1
*Rhodotorula mucilaginosa*	1 (3)	>8	<0.06	1	0.5	0.5	1
Total	30 (100)						

^†^ Two isolates from 2015 did not have susceptibility testing performed, shaded squares indicate that the measured MIC is above the systemic therapeutic levels usually achieved, but no breakpoints are available for topical medication for multiple isolates, the MIC is given as the range of the values.

**Table 4:** Ophthalmic outcomes categorised by causative fungal species.

Clinical Finding	*Fusarium* spp.	*Curvularia* spp.	Other *	Total
Number of patients	9 (29)	12 (39)	10 (32)	31 (100)
**Outcomes**				
Good (%)	5 (55)	3 (33)	5 (83)	13 (54)
Moderate (%)	0	1 (11)	0	1 (4)
Poor (%)	1 (11)	0 (0)	0	1 (4)
Lost to follow-up (%)	3 (33)	5 (56)	1 (17)	9 (38)
Complications				
Corneal scarring (%)	7 (88)	4 (67)	3 (43)	14 (67)
Non healing epithelial defect (%)	1 (13)	0	1 (14)	2 (10)
Corneal perforation (%)	1 (12.5)	0	0	1 (5)
Descemetocele (%)	0	1 (17)	0	1 (5)
Elevated intraocular pressure (%)	1 (13)	0	1 (14)	2 (10)
Corneal transplant (%)	1 (14)	1 (16)	0	2 (10)
Enucleation (%)	0	0	1 (14)	1 (5)

Note: * Not enough data for range of other fungal types.

## Data Availability

The data presented in this study are available on request, subject to review, from the corresponding author due to privacy and ethical reasons.
